# A Video Guide to Reshaping the Breast Through Staged Mastopexy After Nipple-Sparing Mastectomy

**DOI:** 10.1093/asjof/ojaf038

**Published:** 2025-05-07

**Authors:** Kelly A Campbell, Kristen Whalen, Nicole K Le, Madison Tyle, William Nicholas Doyle, William West, Timothy Nehila, Lauren Kuykendall

## Abstract

Nipple-sparing mastectomy (NSM) has gained popularity because of its improved aesthetics and oncological outcomes. This study focuses on staged mastopexy as a technique for optimizing aesthetics in patients with a history of NSM and implant-based reconstruction. The authors aim to evaluate staged mastopexy as a technique to address complications such as implant and nipple–areolar complex malposition, rippling, and ptotic skin envelope, with a focus on patient selection, surgical technique, and patient-reported outcomes. The authors performed a retrospective analysis on a series of 5 patients who underwent NSM and immediate implant-based reconstruction with staged mastopexy by a single surgeon between 2020 and 2023. Descriptive analyses and *t*-tests were used. Significance was defined as *P* ≤ .05. The cohort consisted of 5 patients (10 breasts) with a median age of 43 ± 6.5 years, and average BMI was 22.9 ± 2.2 kg/m^2^. The mean interval from mastectomy to mastopexy was 431.5 ± 232.1 days. Notably, no cases of partial or total nipple necrosis were observed. BREAST-Q surveys revealed significantly higher satisfaction scores for breasts (74 ± 19.9, *P* = .01) compared with normative values, with an 80% response rate. Staged mastopexy demonstrates a safe and reproducible technique for correcting nipple and implant malposition following NSM and implant-based reconstruction.

**Level of Evidence:** 4 (Therapeutic)

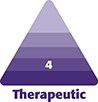

Mastectomies have evolved dramatically to include tissue-sparing approaches for prophylactic and therapeutic purposes. To that end, nipple-sparing mastectomy (NSM) with implant-based reconstruction is increasingly common because of high rates of satisfaction and safe oncologic results in appropriate patients.^1-5^

Anatomic features, including macromastia and breast ptosis, are traditionally contraindications to NSM because of higher rates of complications, including nipple–areolar complex (NAC) and mastectomy flap ischemia, NAC malposition, and overall unaesthetic result.^2,4,6-9^

The purpose of this study is to evaluate a series of patients who underwent NSM with staged mastopexy to mitigate aesthetic complications including implant and NAC malposition, rippling, and ptotic skin envelope in patients who desired to preserve their native NAC. We detail our preoperative patient selection, surgical technique with intraoperative video supplementation, and patient-reported outcomes.

## METHODS

A retrospective case series of patients who underwent NSM and immediate implant-based breast reconstruction with staged mastopexy by a single surgeon between 2020 and 2023 was performed. The patients included were consecutive patients, and there was not an inclusion/exclusion criterion applied. BREAST-Q scores were obtained on the same date for every patient included, August 7, 2023, over the phone with a secure line utilizing the satisfaction with breast, psychosocial well-being, sexual well-being, and physical well-being of the chest domains.

Descriptive analyses and *t*-test were performed. Data were analyzed using SAS 9.4 (SAS Institute, Inc., Cary, NC), accessed from the University of South Florida. Significance was defined as *P* ≤ .05. BREAST-Q scores were compared with published normative scores.^10^

This study was IRB approved by the University of South Florida.

### Description of Surgical Technique

#### Preoperative Markings

Patients are marked preoperatively in the seated position with standard breast markings, including the ideal breast meridian as well as the existing and desired level of the inframammary fold (IMF). The current and ideal lateral breast border is marked if implant repositioning is necessary.

#### Capsule Dissection

An incision is made along the IMF and carried straight down to capsule. If the IMF needs to be elevated, the capsule is dissected inferiorly and mobilized posteriorly off the chest wall to the point of the desired IMF. An anterior capsulotomy is performed near the chest wall at the level of the new IMF position, and the breast implant is removed (see Part 1 of Video which demonstrates preoperative evaluation and capsule dissection).

#### Capsulorrhaphy

If the implant has lateralized or a reduction in implant size is planned, a lateral capsulorrhaphy is performed. This is typically designed based on the anterior axillary line and desired implant base width. When marking the capsulorrhaphy, it is important to keep the nipple centered and elevated at the desired implant projection to prevent nipple lateralization or malposition. The capsule is incised with cautery, and 1 to 2 layers of suture repair are performed with 2-0 PDS (polydioxanone) by Ethicon suture in a running fashion (see Part 2 of Video which demonstrates capsulorrhaphy technique).

An implant sizer is placed in the pocket to confirm appropriate size and positioning. The capsule is then plicated inferiorly to tighten the pocket and suspend the implant in a more superior position. The capsule is secured to the chest wall along the new IMF position using 2-0 PDS. This improves upper pole fullness and reduces rippling (see Part 3 of Video which demonstrates implant sizer placement and capsule repair).

#### Mastopexy

The patient is sat up on the operating room table to 90° with the arms prepped in and draped at the patient's side. The NAC is then marked with a standard 38 or 42 mm cookie cutter, and the skin is tailor tacked to create the desired breast shape based on skin redundancy. Superior nipple position may be limited based on skin suppleness and chest wall anatomy (see Part 4 of Video which demonstrates mastopexy tailor tacking and skin excision).

In the supine position, the marked excess skin is excised full thickness along the IMF. The permanent implant is placed, and the capsulotomy is closed at the desired tension with interrupted 2-0 PDS (see Part 5 of Video which demonstrates capsulotomy and inframammary incision closure). The vertical excess skin is excised down to the level of the subcutaneous fat to maintain central soft-tissue volume. The medial and lateral mastectomy flaps are elevated off the implant capsule to mobilize the vertical limbs for a tension-free closure and allow space for mesh placement, if indicated for additional support (see Part 6 of Video which demonstrates medial and lateral flap elevation).

#### Mesh Placement

When indicated for additional IMF support, an inferior mesh sling is utilized to reinforce the implant position. The lead surgeon initially utilized poly-4-hydroxybterate (P4HB), a bioresorbable polymer commonly used for mesh support, with the rim providing additional inferior support when necessary. Durasorb Monofilament Mesh by Integra, another bioresorbable option, has since been added as an alternative, although P4HB remains the preferred choice. The superior edge of the rim is removed to reduce palpability. The mesh is inset along the inferior border of the capsule, following the contour of the implant using 2-0 PDS. The remaining medial, lateral, and superior edges are inset to the breast capsule, using 3-0 Vicryl (polyglactin 910) or PDS, both by Ethicon, whereas gravity is simulated on the implant to reduce blunting (see Part 6 of Video which demonstrates mesh placement).

#### Closure

The dermis around the NAC can be superficially scored to allow better mobilization for closure. Its vascularity is preserved through the surrounding subdermal plexus. However, if the tissue is thin or there is a history of tissue compromise, the dermis should be left intact. The breast skin is re-draped and closed using 3-0 and 4-0 Monocryl by Ethicon.

#### Postoperative Care

After skin closure is complete, the surgical site is dressed using a topical skin adhesive, followed by paper tape, to ensure secure wound closure and protection. The patient is then placed in a surgical bra with gauze pads for added support. Patients are required to wear bra support for a minimum of 6 weeks up to 3 months. Drains are not typically used during this procedure. Postoperative antibiotics targeted at skin flora are provided for the patient only when mesh is used in addition for 1 week as a preventative measure.

## RESULTS

**Figure 1.**
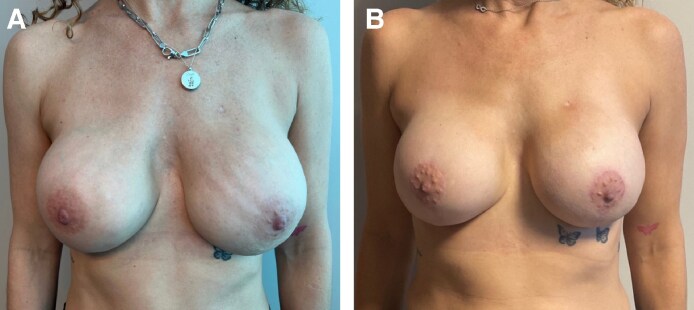
(A) Preoperative photograph of a 49-year-old female who had a history of bilateral nipple-sparing mastectomy with prepectoral implant-based reconstruction (Sientra HSC + high profile, smooth round silicone 505 cc) with acellular dermal matrix who presented 12 months postoperatively with breast ptosis, asymmetry and implant malposition, and rippling. (B) Six-month postoperative photograph following breast revision with wise-pattern mastopexy, implant exchange, capsulorrhaphy, and inferior mesh placement (MENTOR MemoryGel, Irvine, CA; moderate high profile Xtra 500 cc, smooth round silicone).

Five patients (with a total of 10 breasts) underwent this revision technique between 2020 and 2023. Figure 1 depicts the before and after for 1 of these patients. The median age was 43 ± 6.5 years (range, 29-48 years) and average BMI was 22.9 ± 2.2 kg/m^2^ (range, 20.8-27.12 kg/m^2^). All patients were diagnosed with unilateral breast cancer but chose to undergo bilateral mastectomy as a prophylactic measure for the contralateral breast. One patient received neoadjuvant chemotherapy, and another underwent adjuvant chemotherapy. Notably, none of the patients in our cohort received radiation therapy. Four patients had a history of immediate reconstruction with tissue expanders (TEs) (80%) and 1 patient had direct-to-implant reconstruction (20%). The prosthesis was in the prepectoral plane in 3 patients (60%) and the subpectoral plane in 2 patients (40%). The median resected breast tissue weight was 422.5 ± 129.3 g (range, 225-570 g). The average size of implant at initial reconstruction was 570 ± 55.3 cc (range, 385-650 cc), whereas the average implant size at mastopexy was 511 ± 97.4 cc (range, 385-650 cc). The average time from mastectomy to mastopexy was 431.5 ± 232.1 days (range, 176-805 days). There were no complications, including nipple or mastectomy flap ischemia. Only 1 patient (20%) underwent an additional revision with breast fat grafting. The average follow-up time was 129.2 ± 85.2 days (range, 23-237 days).

There was an 80% response rate for the BREAST-Q, with 1 patient not completing the survey. The mean satisfaction with breasts score was 74.3 ± 19.8. The average psychosocial well-being, sexual well-being, and physical well-being of the chest were 74.3 ± 17.8, 58.0 ± 17.8, and 81.3 ± 13.4, respectively. The physical well-being of the chest was lower than normative (81.3 ± 13.4 vs 93 ± 11, *P* < .01); however, our satisfaction with the breast was significantly higher (74.3 ± 19.8 vs 58 ± 18, *P* = .01).^10^ There were no significant differences between the psychosocial well-being (71 ± 18, *P* = .61) and sexual well-being scores (56 ± 18, *P* = .75; Table 1).

**Table 1.** BREAST-Q Scores of Cohort With Normative Values^11^

BREAST-Q module	Mean ± SD	Normative (mean ± SD)	*P*-value
Satisfaction with breasts	74.25 ± 19.80	58 ± 18	.01
Psychosocial well-being	74.25 ± 17.80	71 ± 18	.61
Sexual well-being	58 ± 17.78	56 ± 18	.75
Physical well-being	81.25 ± 13.38	93 ± 11	<.01

SD, standard deviation.

## DISCUSSION

Advances in surgical approaches to breast cancer and reconstruction, including NSM, device placement in the prepectoral plane, use of supportive scaffolds, and fat grafting, have significantly improved aesthetic results for our patients.

Specifically, NSM affords superior aesthetics because of the preservation of the skin envelope and NAC, creating a natural breast mound with central projection.^12^ Implant-based breast reconstruction following NSM can afford highly aesthetic results for patients; however, postoperative nipple and implant malposition remains a challenge.

Strategies to optimize nipple position in implant-based reconstruction after NSM have been proposed. Like other surgical complications, prevention is the best method. To this end, primary reduction mammoplasty with staged NSM can be performed in prophylactic cases but may unnecessarily delay treatment in patients with active cancer.^4,7^ When considering surgical timing, premastectomy mastopexy is generally reserved for cases, where adequate time is available for planning and healing between stages. However, in patients undergoing bilateral mastectomy without the opportunity for a staged approach, postmastectomy mastopexy is preferred. In such cases, the lead surgeon places a TE initially to confirm the patient's desired volume. During the exchange phase, postmastectomy mastopexy is performed alongside capsule adjustments and mesh support to optimize aesthetic outcomes. NSM with skin reduction incisions can similarly improve nipple position during the initial surgery but can lead to ischemic complications.^7,9,13,14^ Secondary NAC repositioning is described but has been largely limited to crescentic or periareolar excisions, which can only correct mild-to-moderate cases of breast ptosis and/or asymmetry.^7,9^ Specifically in implant-based reconstructions, risks associated with this maneuver include NAC necrosis and implant injury or exposure.^9,15^

In addition to nipple malposition, implants can become inferiorly and laterally displaced over time.^16^ This is because of unopposed action of the pectoralis muscle when the implant is subpectoral and because of attenuation of the capsule and acellular dermal matrix/mesh in the case of prepectoral reconstruction. Patients with thin, soft-tissue coverage can develop rippling, which can be exacerbated with increased laxity in the capsule/breast pocket. Appropriate treatment is dependent on the capsule and soft-tissue quality and includes fat grafting, implant exchange, and/or capsulorraphy.^16,17^ In patients with thin mastectomy flaps, the lead surgeon offers fat grafting as an initial step, followed by staged mastopexy 3 to 6 months later to improve candidacy for the procedure. For patients undergoing 2-stage reconstruction with TE to implant, fat grafting is typically performed at the expander exchange phase. This approach ensures adequate soft-tissue bulk during the mastopexy phase, particularly when patients wish to downsize or lift.

We propose a safe and reproducible breast revision method that effectively targets the range of aesthetic complications that can be encountered following NSM, including nipple and implant malposition, rippling, and soft-tissue redundancy.

Following mastectomy, staged mastopexy maintains perfusion of the nipple based on the subdermal plexus while decreasing the skin envelope and repositioning the nipple. Typically, the NAC is left connected with the capsule in a central/superior-type pedicle. The amount of dermal excision/release inferior to the NAC depends on the degree of nipple elevation and the compliance of the tissue following pocket adjustments. This technique is particularly effective for addressing mild degrees of breast ptosis, where conservative elevation of the NAC can be achieved with minimal dermal release, preserving vascularity. However, the lead surgeon has observed that some degree of dermal release is often necessary to achieve sufficient nipple elevation, particularly when the capsule is being tightened or the implant is elevated during the procedure. For small degrees of ptosis, a more conservative approach to dermal preservation can be employed to balance outcomes with vascular safety. We did not experience any ischemic complications with this maneuver because of its performance in a delayed fashion. Full-thickness elevation of the mastectomy flaps off of the capsule, as opposed to a skin-only mastopexy as recommended by the literature in the setting of NSM, helps provide a tension-free closure and allows greater nipple elevation.^9^ An inferolateral capsulorrhaphy reduces the pocket size, which corrects implant malposition and improves rippling. This can be an important tool, especially in the thin population with limited fat grafting donor sites. To prevent recurrent implant and soft-tissue descent, a mesh can be easily placed over the lower pole of the implant because of the ample exposure from mastectomy flap elevation.

We had an 80% response rate to our BREAST-Q survey. The patient who did not complete the BREAST-Q survey was the patient with the shortest follow-up time. The mean satisfaction with breasts, psychosocial well-being, and sexual well-being were higher than normative data. However, physical well-being of the chest was lower than normative data. This is likely because normative values were established through the evaluation of women aged 18 years or older without a history of breast surgery or breast cancer. Having surgery on the chest with associated scar tissue is likely to present with lower chest well-being when compared with women without surgery.

One patient in our series underwent additional revisional surgery with liposuction and fat grafting. Fat grafting has been shown to improve patients’ overall satisfaction with their aesthetic result and should be considered as an adjunct during the staged mastopexy.^5^

Limitations of this study include its retrospective nature and small sample size. Furthermore, a potential criticism is the need for a second-stage operation, but 20% to 40% of women undergo revisional breast reconstruction.^16^ Another limitation of this study is the lack of consistent preoperative and postoperative measurements of the NAC movement. Although this metric was not recorded in this series, it has been incorporated into our ongoing data collection and will be evaluated in future studies to provide more robust outcomes data. With the increasing prevalence of NSM and direct-to-implant reconstruction, knowledge of revisional surgical techniques to improve aesthetics is essential for plastic surgeons.

This technique may also expand the patients who are eligible for NSM to include those with Grade 1 to 2 breast ptosis and mild-moderate excess skin envelopes with the understanding that a staged mastopexy would be required.

## CONCLUSIONS

Staged wise-pattern mastopexy with mesh support is a safe and reproducible method to optimize nipple and implant position after NSM and implant-based reconstruction.
